# Parental Influence in Forming Preschool Children’s Eating Behaviors—A Cross-Sectional Survey in Chongqing, China

**DOI:** 10.3390/healthcare7040140

**Published:** 2019-11-07

**Authors:** Hongmei Hu, Chenlu Yang, Fang Tan, Xin Zhao, Xingxing Du, Jiyu Liang, Tingting Wu, Haozhuo Wang, Zixin Qiu, Hui Zhang, Jiaqiong Zhang, Weiwei Liu

**Affiliations:** 1 College of Pre-school Education, Chongqing University of Education, Chongqing 400067, China; 2 Children’s Research Institute, Chongqing University of Education, Chongqing 400067, China; 3 Chongqing Collaborative Innovation Center for Functional Food, Chongqing University of Education, Chongqing 400067, China; 4 Family Education Guidance Center for 0-6 Years Old, Chongqing University of Education, Chongqing 400067, China; 5 School of Public Health and Management, Chongqing Medical University, Chongqing 400016, China; 6 Department of Public Health, Our Lady of Fatima University, Valenzuela 838, Philippines; 7 Faculty of Teacher Education, Yangtze Normal University, Chongqing 408100, China

**Keywords:** eating behavior, preschool children, parent influence, nutrition, China

## Abstract

Children’s eating habits are closely related to their health problems and the outlook for children’s nutritional statuses appears poor. A cross-sectional survey was conducted among parents of preschool children from December 2018 to January 2019. Sixteen representative kindergartens in 6 districts of Chongqing, China, were included in the study. We took 2200 samples and collected information by questionnaire and after screening, 1781 questionnaires were valid and finally included in the data analysis (*n* = 1781). Ordinal logistic regression analysis found that age, fathers’ education level, forced diet and perception of children’s body shape were factors associated with children’s eating behaviors (ordered logistic regression/three-level eating behavior; odds ratios *p* < 0.05). 80.24% of preschool children may have unhealthy eating behavior in this survey and 80.35% of parents had forced their children to eat. Eating behaviors of preschool children in Chongqing are closely related to family factors. This study provides important insight for parents and health care workers in China to improve preschool-aged children’s nutritional behaviors. Intervention programs should focus on parents with low income, low education levels, coercive dietary behaviors and deviated body shape perceptions to reduce children’s eating behavioral problems.

## 1. Introduction

Lifelong eating habits are associated with early childhood eating behaviors [1]. In early childhood, eating behavior undergoes significant changes. Evidence shows that children’s dietary behaviors are formed at the end of preschool and remain stable after school age [2]. If unhealthy eating behavior goes uncorrected during the critical period when children’s behavioral habits are developing and forming, their lifelong health will be negatively affected [3,4].

Eating behavioral problems mainly include avoidance of food, irritability [5], prolonged eating time and pickiness [6]. In western countries, the incidence of eating behavior problems in children is 30–45% [7]. This number reached 65.1% in China [8].The eating behavior problems of preschool children are closely related to unhealthy outcomes, such as being underweight [5], overweight [5,9], obese [9] or physiologically anorexic [10]. Early childhood nutritional statuses appear poor both in China and worldwide. In 2018, the World Health Organization (WHO) reported that 40 million children under 5 years old were overweight. Since 2000, the number of overweight children under age five in Asia has increased by 33% [11]. In 2015, a report from the China Health Planning Commission (CHPC) revealed that 8.4% of children under 6 years old were overweight.

Factors that may be relevant to children’s eating behavior problems and implementing interventions are being actively explored. Eating behavioral formation of preschool children is closely related to family, society, environment, the media and other factors [12]. Parents play an irreplaceable role in the formation of preschool children’s eating behaviors, as children are good at learning and imitating parental behavior [12,13,14,15]. According to one report, children’s eating behaviors are related to their parents’ knowledge level, feeding style and eating behaviors [13,14,15]. During the preschool period, parents can better develop their children’s nutrition, which is significantly impacted by their eating behavior [16,17,18]. However, at present, parents’ awareness regarding their children’s nutrition in China and worldwide remains relatively weak [19,20]. Thus, parents’ influence on children’s eating behavior has gradually become the focus of attention.

Few studies have been conducted on the correlation between parents’ nutritional knowledge and behavior and children’s eating behavior in China. This study assessed the nutritional status and parental awareness of preschool children in Chongqing and explored the sociodemographics and factors affecting children’s eating behaviors based on their families, especially their parents’ awareness and behaviors. We hope that the development of relevant interventions can be promoted through the exploration of factors related to eating behavior of preschool children.

## 2. Materials and Methods

### 2.1. Method and Participants

A cross-sectional survey was conducted in Jiangbei District, Shapingba District, Nanan District, Banan District, Fuling District and Xiushan County in Chongqing, China, from 15 December 2018 to 15 January 2019. Participants were parents of children from 16 kindergartens in six regions with different levels of economic development. Parents were interviewed face-to-face with questionnaires and all investigators were intern-kindergarten teachers who had received unified training.

### 2.2. Sample

Previous surveys have shown that the incidence of dietary behavioral problems among preschool children in China ranges from 39.7–65.1% [8]. Cluster sampling and the formula, *n* = [Z^2^_1-α/2_ π (1 × π)]/δ^2^ × (1 + 1/2) were used in this study. If α = 0.01, Z_1-α/2_ equals 2.58. The incidence of dietary behavior problems among preschool children was 39.7%, that is, π = 0.397. In this study, δ = 0.05 and *n* = 957. Considering a 10–15% missing rate for unreturned surveys in addition to the sampling error, the minimum sample size was extended to 1100 cases.

In total, 2200 parents participated in the study, with a questionnaire response rate of 86.19% (1896/2200). The questionnaire contained 40 questions. If more than 35 questions were answered, it was regarded as a valid questionnaire. Of the respondents, 1781 answered at least 90% of the questions (36/40) and were finally included in the statistical analysis (*n* = 1781).

### 2.3. Ethical Approval

All subjects provided informed consent before participating in the study. The ethical approval number of this study is from Chongqing Collaborative Innovation Center for Functional Food in Chongqing University of Education (201901HS01).

### 2.4. Reliability and Validity

A panel of experts in nutrition, epidemiology and target populations established the questionnaires on the basis of consulting papers and Chinese Dietary Guidelines. Two months before the formal survey, a pilot survey was conducted at a teacher recruitment fair. Approximately 90 datapoints were collected and the questionnaire was subsequently modified based on these results. Reliability and validity tests were applied. The reliability of Cronbach’s Alpha coefficient was 0.811, using Kaiser-Meyer-Olkin’s validity statistical test (KMO = 0.775) and Bartlett’s sphericity test (*p* < 0.0001).

### 2.5. Questionnaire

#### 2.5.1. Demographics

The demographic characteristics included the child’s age, height, weight, sex and number of siblings; the parents’ height, weight, education level and occupation; whether the home was single-parent (yes/no); and monthly household income. Of all samples, the mean age of the 1781 children enrolled in the survey was 5.27 years and 928 were boys. More than half of the respondents had only one child (no siblings; 58.06%).

#### 2.5.2. Parents’ Nutrition Opinions

Seven nutrition-related questions were included, which mostly used five-level scoring—(1) frequency of forcing the child to eat foods they dislike (2) parents’ cognition of their child’s body shape (very thin/slender/average/overweight/obese), (3) parents’ attitudes and behaviors after learning their child was overweight, (4) parents’ opinions on obesity factors (excessive fat intake/excessive sugar intake/eating speed/too little exercise/genetic factors/unsure), (5) recognition of the need for early prevention of obesity in children (yes, we should start prevention now/yes but too early/no), (6) frequency of teaching the child about a healthy diet and (7) parents’ use of food to motivate their child (sweet drinks/fast food/fried food/fruit/candy/no use of food incentives). According to our survey, 80.35% of parents reported that they have forced their children to eat foods the children disliked, while 59.40% of parents had inaccurate perceptions of their children’s body shape.

#### 2.5.3. Children’s Eating Behavior

Sixteen questions were included on the children’s eating behaviors as follows—food preferences (meat/vegetarian/non-preferential), taste preferences (sweet/spicy/sour/salty /bitter/other), eating behavioral problems (disruption or getting angry/no interest/fear of certain foods/watch TV/play with toys/be dazed/eating foods the child does not like angers them or they spit it out/these have never happened), time taken to eat (within 10 min/10–30 min/more than 30 min), eating healthy foods (yes/no), frequencies of eating the following foods or behaviors within a week—breakfast, eggs, soy products, fruits, nuts (peanuts, almonds, walnuts), seafood, meat (pork, beef and poultry), dairy products, carbonated beverages, fast food, fried foods (almost every day/4–5 times per week/2–3 times per week/ ≤ once per week). For the child’s dietary behavior, each option was given a specific score. When the total score is less than 60% of the full score (less than 28 points), it is considered low level. The total score is between 75% and 60% of the full score (29–35 points), which is considered to be medium level. When the total score is greater than 75% of the full score (35–47 points), it is considered to be high level and high level means relatively positive eating behavior.

### 2.6. Data Analysis

All data were double-entered using Microsoft Office Excel 2007 (Microsoft, Redmond, Washington, DC, USA) All statistical data were analyzed using two-sided *t*-tests in SAS statistical software (SAS, version 9.4; SAS Institute, Cary, NC, USA). Statistical significance was set at *p* < 0.05. In the descriptive analysis, the characteristics of the participant’s children were summarized using the mean and standard deviation or frequency and percentage. Variance analysis was used to test the significance of the differences between continuous variables. The variability of the categorical variables was tested using the chi-square test. Logistic regression analysis was used to evaluate the correlation between factors and children’s eating behavior.

Multivariable analysis was conducted on the factors influencing children’s eating behavior. Height and weight are self-reported in the questionnaire and body mass index (BMI) = weight (kg)/height^2^ (cm). The calculated BMI was compared with a Chinese comparison of the mature BMI values of sex and age of children [21,22], which was used to judge the body shape of children. Parents’ perceptions of children’s body shapes were classified as conforming or not to their child’s body shape. 

## 3. Results

### 3.1. Demographics of the Children

Table 1 shows that most parents had completed higher education. The monthly income of most families ranged from 560–1120 dollar (30.88%). Variance analysis showed that parental occupation (for fathers’ occupation, *p* = 0.0053 and for mothers’ occupation, *p* = 0.002), parents’ educational level, whether they were single-parent families or not and the family’s monthly income were significantly correlated with children’s dietary behavior scores (*p* < 0.001). Relevance analysis showed that BMI of mothers (*p* = 0.001) and age of children (*p* = 0.003) may be negatively correlated with children’s dietary behavior score. There was no significant difference between father’s BMI, the number of siblings and breastfeeding time and children’s dietary behavior score.

### 3.2. Parents’ Opinions of Nutrition and Children’s Eating Behavior

Univariate analysis was conducted between each variable and eating behavior score (Table 2). The results showed that there was a significant correlation between compulsory eating behavior, parents’ perception of children’s body shape, parents’ nutritional knowledge and nutritional education and children’s eating behavior score.

Table 3 shows that approximately 53.74% of children may have preferential eating behaviors, 57.78% ate their meals in 10–30 min, 8.15% did not eat breakfast every day. Chinese dietary guidelines point out that preschool children should eat cereals, eggs, fruits, vegetables, fish, lean meat, soy products and dairy products every day, however, 35.36% consumed eggs every day and more than 40% consumed soy products 2–3 times per week. Less than 60% of children ate fruit, meat or dairy products every day. The most common dietary problem for children was watching TV at meals (51.54%) and 80.24% of preschool children may have unhealthy eating behavior in this survey.

### 3.3. Logistic Regression Model for Factors Influencing Eating Behaviors at Different Levels

Children whose fathers had only basic education were more likely to present low-level eating behaviors than were those whose fathers had a higher education (OR: 1.875; 95% confidence interval (CI): 1.220–2.882). Children who had occasionally been forced to eat foods they disliked were more likely to show low level eating behavior compared with those who were never forced (OR: 0.463; 95% CI: 0.317–0.675) and those whose parents had less objective cognition of their child’s body shape were more likely to show low level eating behaviors compared with those whose parents had an objective cognition (OR: 0.755; 95% CI: 0.574–0.993).

A high possibility of medium-level eating behavior was observed for children who had never been forced to eat foods they disliked compared with those who occasionally been forced (OR: 2.0903; 95% CI: 1.429–3.057). A high possibility of high-level eating behavior was observed in children whose parents had an objective cognition of their children’s body shape compared those whose parents had a less objective cognition (OR: 2.719; 95% CI: 1.084–6.815; Table 4).

### 3.4. Ordered Multivariate Logistic Regression Factors Influencing Children’s Eating Behavior

The children’s eating behavior was scored according to the scoring criteria in Table 5 and was used as a dependent variable in further analyses. To further investigate the factors that affect the characteristics of children’s eating behavior, we chose the following parameters—child’s age, BMI and sex; parents’ BMI, education level and occupation; single-parent home (yes/no), monthly household income, frequency of forcing children to eat foods they dislike (always/often/occasionally/seldom/never), whether the parents had an objective cognition of children’s body shape (yes/no), whether the parents thought childhood obesity required early prevention (yes, we should start prevention now/yes but it is too early/no) and frequency of teaching children about a healthy diet (always/often/occasionally/seldom/never).

In the ordinal logistic regression analysis model, partial regression coefficient (β) = estimate. Children’s age and eating behavior score were negatively correlated (95% CI: −0.2949–(−0.0521); *p* = 0.0051). Children whose fathers had only a basic education level were less likely to have high eating behavior scores than were those whose fathers had a higher education level (95% CI: −0.6728–(−0.1905); *p* = 0.0005). Compared with children who were occasionally forced to eat foods they disliked, children who were never forced were more likely to have high eating behavior scores (95% CI: 0.1416–0.7565; *p* = 0.0417). Compared with children who were only occasionally forced to eat foods they disliked, children who had often been forced were less likely to report high eating behavior scores (95% CI: −0.1927–(−0.1625); *p* = 0.0082). Parents with objective cognition of children’s body shapes were more likely to report high eating behavior scores (95% CI: 0.0229–0.2926; *p* = 0.0218; Table 6).

## 4. Discussion

In 1985, a study first pointed out that 2–5 years of age is the key period in which children should develop good eating behaviors [23]. In this study, 80.24% of preschool children may have unhealthy eating behavior. Eating behavior problems mainly include pickiness, preference, unhealthy eating behavior, food avoidance and boredom [5,6]. Due to the lack of statistical cut-off value, the prevalence of eating behavior problems in China ranges from 35–65.1% [8]. It was reported that the prevalence of eating behavior problems of preschool children reached 79.6% in Chengdu, China [24], which is similar to our study. Chongqing and Chengdu are close in geographical location and similar in diet pattern.

Previous studies have shown that preschool children’s eating behavior is related to kindergarten teachers’ education, kindergarten environment [25], peers [26], parents’ cognition and behavior [13,14,15] and children’s physical activity [27]. Parents play an irreplaceable role in forming their children’s dietary behavior [12,13,14,15]; therefore, we focused on the correlation between parental awareness and children’s eating behavior.

The main findings were that children whose fathers had higher education were more likely to display more positive eating behaviors. Previous studies have shown a correlation between children’s eating behavior and parents’ educational level [28,29] but this may have been due to small sample sizes. We found no clear link with mothers’ educational levels. Another possible factor associated with higher eating scores was parents’ objective cognition of their child’s body shape. We found that children’s eating behavior was related to their mother’s occupation. Children of mothers with professional job tend to have higher eating behavior scores. This may be due to the fact that mothers with this career can provide more health care for their children [30]. In addition, children’s age was negatively correlated with eating behavior score. Regarding the negative correlation between age and eating behavior score, we infer that this may be related to increased emotional overeating with age [31].

Parents’ perception of children’s body shape is also a significant correlative factor with children’s eating behavior found in this study. However, in this study, nearly 60% of parents have biased perceptions of their children’s body shape. Regarding the importance of parents’ perceptions of children’s body shape, one study suggested that this is related to parents’ weight restrictions on children [32], which may be related to their child’s eating behavior score.

We also found that children who were not forced to eat were associated with higher dietary eating scores. However, more than 80% of parents reported that they forced their children to eat foods the children disliked. Forcing children to eat is more likely to lead to eating behavior problems and reduce the diversity in children’s diets. A study in Sweden also showed that parents paid attention to children’s diet during meals, making the children more reluctant to eat [33]. Numerous studies have shown that how parents feed their children can affect the children’s diets [34,35,36,37]. During feeding, parents control the children’s diet, including the type and amount of food. According to these studies, parental intervention in children’s diets is associated with lower eating behavior scores for the following reasons—(1) The use of stress to force children to eat is negatively correlated with children’s food acceptance [34,35]; (2) Stress can negatively affect children’s emotional response and healthy food intake [36]; (3) Parents’ restrictions on children’s diets may impair their child’s ability to self-regulate their food intake [37].

Several studies have shown that parents’ eating habits are closely related to their children’s eating behavior [2,12,38,39,40], which has both positive and negative effects. The specific influencing mechanism that parental eating behavior has on preschool children’s eating behavior remains unclear. Study suggests that this may be due to family dietary habits or emotional transmission [38]. Therefore, the best way to change children’s eating behavior is to change their parents’ eating habits. Simply forcing children to eat is counterproductive. Parents should understand eating behavior and improve their own eating habits to reduce the incidence of children’s eating behavioral problems.

Many factors affect the eating behaviors of preschool children in Chongqing, such as children’s age, father’s education level, mother’s occupation, forced eating behavior and parents’ perception of children’s body shape and so on. Logistic regression analysis showed significant differences in children’s eating behavior and parental factors. Further research should expand the sample population. Health workers must fully consider the factors affecting children’s eating behavior when formulating intervention programs for children’s nutritional behavior.

### Limitation

The study has three limitations. Firstly, the interviewees in this study are parents but there is no question of whether the interviewees are “caregivers,” so there may be information bias. Secondly, only parents’ nutritional knowledge and behavior were investigated but insufficient questions about family dietary environment were not designed. Reduction in the frequency of certain foods consumed due to allergies was not taken into account. Thirdly, cross-sectional surveys are not very good at presenting causality. As the questionnaire is self-designed, it may only be applicable to areas with similar economic development level in Chongqing, China. These will be revised and improved in the future research.

## 5. Conclusions

The eating behaviors of preschool children in Chongqing are closely related to family factors, such as children’s age, father’s education level, mother’s occupation, forced eating behavior and parents’ perception of children’s body shape and so on. This study provides important insight for parents and health care workers in China to improve nutritional behavior in preschool-aged children. Intervention programs should focus on parents with low income, low educational levels, dietary coercive behavior and a deviated perception of body shape to reduce children’s eating behavioral problems.

## Figures and Tables

**Table 1:** Characteristics of the study sample.

Categorical Variables	Overall Sample *n* (%)	Mean (SD) of Eating Behavior Score	*p*-Value ^a^
Child Gender			0.6175
Male	938 (52.67%)	31.58 (5.92)	
Female	831 (46.66%)	31.44 (5.80)	
Missing	12 (0.67%)	31.67 (3.35)	
Number of Sibling			0.2605
0	1034 (58.06%)	31.67 (5.82)	
1	679 (38.12%)	31.35 (5.99)	
More than 1	49 (2.75%)	30.85 (5.12)	
Missing	19 (1.07%)	30.42 (3.87)	
The Duration of Breast-feeding			0.4917
1–3 months	320 (17.97%)	31.36 (5.79)	
4–6 months	330 (18.53%)	31.90 (6.00)	
7–9 months	467 (26.22%)	32.00 (5.61)	
10–12 months	418 (23.47%)	30.84 (5.90)	
More than 12 months	204 (11.45%)	31.65 (5.94)	
Missing	42 (2.36%)	30.33 (6.09)	
Father’s Education Level			<0.0001
Basic education	495 (27.79%)	29.52 (5.78)	
Secondary education	373 (20.94%)	30.89 (5.47)	
Higher education	891 (50.03%)	32.90 (5.69)	
Missing	22 (1.24%)	30.86 (5.31)	
Mother’s Education Level			<0.0001
Basic education	518 (29.08%)	29.84 (5.73)	
Secondary education	373 (20.94%)	30.76 (5.43)	
Higher education	857 (48.13%)	32.87 (5.81)	
Missing	33 (1.85%)	30.94 (5.13)	
Father’s Occupation			0.0053
Leaders	53 (2.98%)	33.43 (6.19)	
Farmers	24 (1.35%)	27.42 (6.97)	
Labors	290 (16.28%)	30.80 (5.70)	
Office staff	266 (14.93%)	32.66 (5.49)	
Commercial/service staff	621 (34.87%)	30.90 (5.87)	
Professional job	143 (8.03%)	33.07 (5.75)	
Others	297 (16.68%)	31.72 (5.64)	
Missing	87 (4.88%)	31.47 (5.98)	
Mother’s Occupation			0.0002
Leaders	20 (1.12%)	34.00 (6.32)	
Farmers	24 (1.35%)	28.29 (6.28)	
Labors	70 (3.93%)	28.91 (5.21)	
Office staff	380 (21.34%)	32.30 (5.81)	
Commercial/service staff	241 (13.53%)	30.70 (5.79)	
Professional job	207 (11.62%)	33.30 (5.44)	
Others	738 (41.44%)	31.21 (5.76)	
Missing	101 (5.67%)	31.14 (6.17)	
Single Parent Home			<0.0001
Yes	1537 (86.30%)	31.67 (5.84)	
No	100 (5.61%)	30.15 (5.47)	
Missing	144 (8.09%)	30.78 (6.02)	
Monthly Household Income ($)			<0.0001
<560	191 (10.73%)	29.31 (6.36)	
560–1120	550 (30.88%)	30.62 (5.66)	
1121–1680	513 (28.80%)	31.91 (5.76)	
1681–2440	202 (11.34%)	32.96 (5.24)	
2441–2800	146 (8.20%)	33.15 (5.75)	
>2800	117 (6.57%)	33.26 (5.68)	
Missing	62 (3.48%)	31.06 (5.51)	
Continuous Variables	Overall Sample Mean (SD)	r	*p*-Value ^b^
Child Age (years)	5.27 (1.06)	−0.18716	0.0003
Father BMI (kg/m^2^)	23.24 (3.00)	0.01676	0.5044
Mother BMI (kg/m^2^)	21.13 (2.61)	−0.29677	0.0001

Note: Education level was categorized as: (1) basic education including primary school and junior middle school; (2) secondary education including senior high school, vocational/technical, secondary school and junior college; (3) higher education including senior college, university, graduate and above. Occupations were categorized as: leaders, farmers, labors, office staff (including government employee and private employee), commercial/service staff or professional job (including teachers and medical staff). SD—standard deviation, and Statistical significance was *p* < 0.05. ^a^ Assessed by variance. ^b^ Assessed by Spearman’s correlation.

**Table 2:** Parents’ opinions of nutrition.

Variables	Overall Sample *n* (%)	Mean (SD) of Eating Behavior Score	*p*-Value ^a^
Frequency of Forcing Children to Eat Foods They Don’t Like			<0.0001
Always	19 (1.07%)	30.26 (5.45)	
Often	125 (7.02%)	29.81 (6.51)	
Occasionally	767 (43.06%)	31.18 (5.82)	
Seldom	520 (29.20%)	31.92 (5.73)	
Never	326 (18.30%)	32.23 (5.75)	
Missing	24 (1.35%)	33.54 (4.40)	
Is Parents’ Cognition of Children’s Body Shape Objective?			0.0011
Yes	720 (40.43%)	32.06 (5.82)	
No	1058 (59.40%)	31.14 (5.85)	
Missing	3 (0.17%)	33.00 (0.82)	
Do Parents Start to Worry About and Adjust Diet, If They Think Their Child is Overweight or Obese?			0.0562
Not worry	473 (26.56%)	31.12 (5.94)	
A little worry but not limit diet	641 (35.99%)	31.78 (5.82)	
Worry about it and limit diet	548 (30.77%)	31.65 (5.67)	
Missing	119 (6.68%)	31.02 (6.39)	
Does Childhood Obesity Need Early Prevention?			0.0013
Yes, we should start prevention now	1138 (63.90%)	31.90 (5.75)	
Yes but it’s too early	464 (26.05%)	31.06 (5.55)	
No	148 (8.31%)	30.42 (6.94)	
Missing	31 (1.74%)	29.22 (6.54)	
Frequency of Teaching Children About Healthy Diet			<0.0001
Always	142 (7.97%)	32.53 (6.51)	
Often	753 (42.28%)	32.20 (6.08)	
Occasionally	647 (36.33%)	30.97 (5.39)	
Seldom	215 (12.07%)	30.39 (5.43)	
Never	18 (1.01%)	28.06 (6.15)	
Missing	6 (0.34%)	30.67 (4.99)	
Parents Usually Use as Incentives to Motivate Their Child **			
Sweet drinks	219 (12.30%)		
Fast food	139 (7.80%)		
Fired food	74 (4.15%)		
Fruit	380 (21.34%)		
Candy	519 (29.14%)		
No use of food incentives	725 (40.71%)		
Factors of Obesity **			
Excessive fat intake	832 (46.72%)		
Excessive sugar intake	991 (55.64%)		
Eating speed	172 (9.66%)		
Too little exercise	906 (50.87%)		
Genetic factors	473 (26.56%)		
Unclear	205 (11.51%)		

SD—standard deviation, and Statistical significance was *p* < 0.05. ^a^ Assessed by variance. **Multiple-choice questions.

**Table 3:** Children’s eating behaviors.

Variables	Low Level *n* (%)	Medium Level *n* (%)	High Level *n* (%)	Overall Sample *n* (%)
Child’s Preferences for Food				
Meat Food	15 (0.84%)	280 (15.72%)	241 (13.53%)	536 (30.09%)
Vegetarian Diet	23 (1.29%)	232 (13.03%)	166 (9.32%)	421 (23.64%)
Non-preferential	3 (0.17%)	174 (9.77%)	621 (34.87%)	798 (44.81%)
Missing	2 (0.11%)	14 (0.79%)	10 (0.56%)	26 (1.46%)
Time for Child to Eat				
Within 10 Minutes	14 (0.79%)	112 (6.29%)	69 (3.87%)	195 (10.95%)
10–30 Minutes	6 (0.34%)	364 (20.44%)	839 (47.11%)	1029 (67.89%)
More than 30 Minutes	22 (1.24%)	217 (12.18%)	124 (6.96%)	363 (20.38%)
Missing	1 (0.06%)	7 (0.39%)	6 (0.34%)	14 (0.79%)
Eating health products				
Yes	6 (0.34%)	122 (6.85%)	362 (20.32%)	490 (27.51%)
No	37 (2.08%)	578 (32.45%)	676 (37.96%)	1291 (72.49%)
Frequencies of Having Breakfast in a Week				
Almost every Day	29 (1.63%)	577 (32.40%)	966 (54.24%)	1572 (88.27%)
4–5 Times	5 (0.28%)	48 (2.70%)	38 (2.13%)	91 (5.11%)
2–3 Times	3 (0.17%)	31 (1.74%)	6 (0.34%)	40 (2.25%)
≦1 Time	2 (0.11%)	8 (0.45%)	4 (0.22%)	14 (0.78%)
Missing	4 (0.22%)	36 (2.02%)	24 (1.35%)	64 (3.59%)
Frequencies of Eating Eggs in a Week				
Almost every Day	6 (0.34%)	118 (6.63%)	504 (28.30%)	628 (35.27%)
4–5 Times	5 (0.28%)	134 (7.52%)	232 (13.03%)	371 (20.83%)
2–3 Times	12 (0.67%)	296 (16.62%)	238 (13.36%)	546 (30.65%)
≦1 Time	16 (0.90%)	140 (7.86%)	61 (3.42%)	217 (12.18%)
Missing	4 (0.22%)	12 (0.67%)	3 (0.17%)	19 (1.07%)
Frequencies of Eating Soy Products in a Week				
Almost every Day	2 (0.11%)	64 (3.59%)	246 (13.81%)	312 (17.52%)
4–5 Times	13 (0.73%)	74 (4.15%)	189 (10.61%)	265 (14.88%)
2–3 Times	20 (1.12%)	284 (15.95%)	441 (24.76%)	738 (41.44%)
≦1 Time	2 (0.11%)	263 (14.77%)	149 (8.37%)	432 (24.25%)
Missing	6 (0.34%)	15 (0.84%)	13 (0.73%)	34 (1.91%)
Frequencies of Eating Fruits in a Week				
Almost every Day	9 (0.51%)	241 (13.53%)	724 (40.65%)	974 (54.69%)
4–5 Times	9 (0.51%)	208 (11.68%)	196 (11.01%)	413 (23.19%)
2–3 Times	10 (0.56%)	177 (9.94%)	95 (5.33%)	282 (15.83%)
≦1 Time	14 (0.79%)	60 (3.37%)	11 (0.62%)	85 (4.77%)
Missing	1 (0.06%)	14 (0.79%)	12 (0.67%)	27 (1.52%)
Frequencies of Eating Nuts in a Week				
Almost every Day	1 (0.06%)	29 (1.63%)	194 (10.89%)	224 (12.58%)
4–5 Times	4 (0.22%)	83 (4.66%)	195 (10.95%)	282 (15.83%)
2–3 Times	9 (0.51%)	256 (14.37%)	441 (24.76%)	706 (39.64%)
≦1 Time	26 (1.46%)	316 (17.74%)	199 (11.17%)	541 (30.38%)
Missing	3 (0.17%)	16 (0.90%)	9 (0.51%)	28 (1.57%)
Frequencies of Eating Seafood in a Week				
Almost every Day	0 (0.00%)	32 (1.80%)	128 (7.19%)	160 (8.98%)
4–5 Times	3 (0.17%)	79 (4.44%)	158 (8.87%)	240 (13.48%)
2–3 Times	9 (0.51%)	240 (13.48%)	462 (25.94%)	711 (39.92%)
≦1 Time	30 (1.68%)	339 (19.03%)	281 (15.78%)	650 (36.50%)
Missing	1 (0.06%)	10 (0.56%)	9 (0.51%)	20 (1.12%)
Frequencies of Eating Meat in a Week				
Almost every Day	11 (0.62%)	316 (17.74%)	727 (40.82%)	1054 (59.18%)
4–5 Times	7 (0.39%)	178 (9.99%)	223 (12.52%)	408 (22.91%)
2–3 Times	13 (0.73%)	144 (8.09%)	78 (4.38%)	235 (13.19%)
≦1 Time	11 (0.62%)	49 (2.75%)	9 (0.51%)	69 (3.87%)
Missing	1 (0.06%)	13 (0.73%)	1 (0.06%)	15 (0.85%)
Frequencies of Having Dairy Products in a Week				
Almost every Day	9 (0.51%)	298 (16.73%)	719 (40.37%)	1026 (57.61%)
4–5 Times	8 (0.45%)	148 (8.31%)	184 (10.33%)	340 (19.09%)
2–3 Times	12 (0.67%)	152 (8.53%)	95 (5.33%)	259 (14.54%)
≦1 Time	9 (0.51%)	92 (5.17%)	37 (2.08%)	137 (7.69%)
Missing	5 (0.28%)	11 (0.62%)	3 (0.17%)	19 (1.07%)
Frequencies of Having Carbonated Beverages in a Week				
Almost every Day	6 (0.34%)	37 (2.08%)	32 (1.80%)	75 (4.21%)
4–5 Times	8 (0.45%)	49 (2.75%)	45 (2.53%)	102 (5.73%)
2–3 Times	5 (0.28%)	83 (4.66%)	81 (4.55%)	169 (9.49%)
≦1 Time	11 (0.62%)	474 (26.61%)	852 (47.84%)	1337 (75.07%)
Missing	13 (0.73%)	57 (3.20%)	28 (1.57%)	98 (5.50%)
Frequencies of Having Fast Food in a Week				
Almost every Day	4 (0.22%)	18 (1.01%)	17 (0.95%)	39 (2.19%)
4–5 Times	7 (0.39%)	58 (3.26%)	27 (1.52%)	92 (5.17%)
2–3 Times	5 (0.28%)	65 (3.65%)	83 (4.66%)	153 (8.59%)
≦1 Time	12 (0.67%)	512 (28.75%)	892 (50.08%)	1416 (79.50%)
Missing	15 (0.84%)	47 (2.64%)	19 (1.07%)	81 (4.55%)
Frequencies of Having Fired Food in a Week				
Almost every Day	4 (0.22%)	19 (1.07%)	19 (1.07%)	42 (2.36%)
4–5 Times	8 (0.45%)	53 (2.98%)	32 (1.80%)	93 (5.22%)
2–3 Times	7 (0.39%)	89 (5.00%)	119 (6.68%)	215 (12.07%)
≦1 Time	14 (0.79%)	498 (27.96%)	852 (47.84%)	1364 (76.59%)
Missing	10 (0.56%)	41 (2.30%)	16 (0.90%)	67 (3.76%)
Children’s Eating Behavior Problems **				
Disruption or Get Angry	10	135	130	275 (15.44%)
Without Interest, Fear of Certain Foods	8	166	120	294 (16.51%)
Watch TV	29	409	480	918 (51.54%)
Play with Toys, be dazed	15	147	142	304 (17.07)
Spit out	10	190	185	385 (21.62%)
These Never Happened	6	80	266	352 (19.76%)

Note: **Multiple-choice questions.

**Table 4:** Odds ratios (ORs) and 95% confidence intervals (CIs) for factors influencing different levels of eating behavior.

Parameter		Low Level				Medium Level				High Level			
		OR	95%CI		*p*-Value	OR	95%CI		*p*-Value	OR	95%CI		*p*-Value
Child Gender	Female vs. Male	1.198	0.923	1.556	0.1745	0.851	0.656	1.105	0.2269	0.990	0.426	2.296	0.9805
Father’s Education Level	Basic education vs. Higher education	1.875	1.220	2.882	0.0014	0.603	0.393	0.926	0.0177	0.347	0.089	1.343	0.0120
	Secondary education vs. Higher education	1.056	0.706	1.580	0.1405	0.883	0.590	1.322	0.4620	2.264	0.515	9.964	0.0358
Mother’s Education Level	Basic education vs. Higher education	1.158	0.752	1.785	0.9176	0.852	0.554	1.312	0.5854	0.899	0.199	4.064	0.3349
	Secondary education vs. Higher education	1.291	0.853	1.954	0.3060	0.890	0.587	1.348	0.8343	0.252	0.065	0.978	0.0123
Monthly Household Income ($)	<560 vs. 560–1120	1.323	0.849	2.063	0.0122	0.751	0.483	1.168	0.0154	1.055	0.278	3.998	0.9079
	1121–1680 vs. 560–1120	0.745	0.533	1.043	0.4596	1.382	0.986	1.936	0.2536	0.995	0.379	2.612	0.9051
	1681–2240 vs. 560–1120	0.611	0.377	0.992	0.1289	1.486	0.917	2.408	0.2470	>999.999	<0.001	>999.999	0.9178
	2241–2800 vs. 560–1120	0.705	0.415	1.199	0.4782	1.303	0.766	2.215	0.6646	2.679	0.300	23.945	0.9542
	>2800 vs. 560–1120	0.724	0.395	1.329	0.6083	1.391	0.755	2.562	0.5232	1.261	0.135	11.797	0.9169
Frequency of Forcing Children to Eat Foods They Don’t Like	Always vs. Occasionally	0.467	0.129	1.684	0.4004	1.749	0.498	6.143	0.5626	>999.999	<0.001	>999.999	0.9534
	Often vs. Occasionally	1.389	0.810	2.381	0.0074	0.712	0.417	1.215	0.0121	1.002	0.185	5.416	0.9489
	Seldom vs. Occasionally	0.656	0.481	0.896	0.5853	1.442	1.056	1.968	0.5600	1.683	0.586	4.838	0.9600
	Never vs. Occasionally	0.463	0.317	0.675	0.0205	2.090	1.429	3.057	0.0134	1.391	0.423	4.574	0.9559
Is Parents’ Cognition of Children’s Body Shape Objective?	Yes vs. No	0.755	0.574	0.993	0.0446	1.193	0.906	1.570	0.2087	2.719	1.084	6.815	0.0329

Note: OR—odds ratio and 95% CI—95% confidence intervals.

**Table 5:** Children’s eating behavior scores.

Subject	Option	Score
Child’s Preferences for Food	Meat Food	0
	Vegetarian Diet	0
	Non-preferential	4
Children’s Eating Behavior Problems	Disruption or Get Angry	Each option is selected with a deduction of one point
	Without Interest, Fear of Certain Foods	
	Watch TV	
	Play with Toys	
	Spit out	
	These Never Happened	6
Time for Child to Eat	Within 10 Minutes	0
	10–30 Minutes	4
	More than 30 Minutes	0
Child’s Behavior of Eating Health Products	Yes	4
	No	0
Frequencies of Having Breakfast in a Week	Almost every Day	4
	4–5 Times	3
	2–3 Times	2
	1 Time	1
Frequencies of Eating Eggs in a Week	Almost every Day	4
	4–5 Times	3
	2–3 Times	2
	≦1 Time	1
Frequencies of Eating Soy Products in a Week	Almost every Day	4
	4–5 Times	3
	2–3 Times	2
	≦1 Time	1
Frequencies of Eating Fruits in a Week	Almost every Day	4
	4–5 Times	3
	2–3 Times	2
	≦1 Time	1
Frequencies of Eating Nuts in a Week	Almost every Day	4
	4–5 Times	3
	2–3 Times	2
	≦1 Time	1
Frequencies of Eating Aquatic Products in a Week	Almost every Day	4
	4–5 Times	3
	2–3 Times	2
	≦1 Time	1
Frequencies of Eating Meat in a Week	Almost every Day	4
	4–5 Times	3
	2–3 Times	2
	≦1 Time	1
Frequencies of Having Dairy Products in a Week	Almost every Day	4
	4–5 Times	3
	2–3 Times	2
	≦1 Time	1
Frequencies of Having Carbonated Beverages in a Week	Almost every Day	1
	4–5 Times	2
	2–3 Times	3
	≦1 Time	4
Frequencies of Having Fast Food in a Week	Almost every Day	1
	4–5 Times	2
	2–3 Times	3
	≦1 Time	4
Frequencies of Having Fired Food in a Week	Almost every Day	1
	4–5 Times	2
	2–3 Times	3
	≦1 Time	4

**Table 6:** Ordered multivariate logistic regression for children’s eating behavior.

Parameter		Estimate	SE	95%CI		*p*-Value
						0.4214
Intercept 2		4.1961	0.8411	2.5476	5.8445	<0.0001
Age		−0.1735	0.0619	−0.2949	−0.0521	0.0051
Child BMI		−0.00765	0.0203	−0.0474	0.0321	0.7059
Gender	Female	−0.0856	0.0654	−0.2137	0.0425	0.1901
	Male (ref.)					
Father BMI		0.000455	0.0204	−0.0395	0.0405	0.9822
Mother BMI		0.0174	0.0226	−0.0270	0.0617	0.4429
Father’s Education Level	Basic education	−0.4316	0.1230	−0.6728	−0.1905	0.0005
	Secondary education	0.2150	0.1156	−0.0116	0.4417	0.0629
	Higher education (ref.)					
Mother’s Education Level	Basic education	0.0176	0.1215	−0.2205	0.2558	0.8845
	Secondary education	−0.1714	0.1163	−0.3994	0.0566	0.1406
	Higher education (ref.)					
Father’s occupation	Leaders	0.1752	0.4038	−0.6163	0.9667	0.6645
	Professional job	−0.0343	0.2973	−0.6171	0.5484	0.9081
	Labors	−0.3285	0.2294	−0.7782	0.1212	0.1522
	Office staff	0.0350	0.2495	−0.4540	0.5240	0.8885
	Farmers	0.1787	1.0119	−1.8046	2.1621	0.8598
	Others	0.2406	0.2398	−0.2294	0.7105	0.3157
	Commercial/service staff (ref.)					
Mother’s Occupation	Leaders	−0.2969	0.6193	−1.5108	0.9169	0.6316
	Professional job	0.7216	0.2877	0.1577	1.2855	0.0121
	Labors	−0.1838	0.3299	−0.8304	0.4627	0.5773
	Farmers	−0.4012	1.0502	−2.4596	1.6572	0.7025
	Commercial/service staff	−0.0297	0.2532	−0.5260	0.4666	0.9067
	Others	0.2714	0.2323	−0.1839	0.7268	0.2427
	Office staff (ref.)					
Single Parent Home	Yes	0.2203	0.1404	−0.0548	0.4954	0.1165
	No (ref.)					
Monthly Household Income ($)	<560	−0.4350	0.1841	−0.7959	−0.0741	0.0182
	1121–1680	0.0560	0.1303	−0.1993	0.3114	0.6672
	1681–2240	0.3082	0.1944	−0.0729	0.6892	0.1129
	2241–2800	0.1726	0.2136	−0.2460	0.5912	0.4189
	>2800	0.1079	0.2454	−0.3731	0.5889	0.6602
	560–1120 (ref.)					
Frequency of Forcing Children to Eat Foods They Don’t Like	Always	0.5012	0.5153	−0.5088	1.5113	0.3307
	Often	−0.6276	0.2373	−1.0927	−0.1625	0.0082
	Seldom	0.0866	0.1739	−0.2542	0.4274	0.6185
	Never	0.3856	0.1893	0.0146	0.7565	0.0417
	Occasionally (ref.)					
Is Parents’ Cognition of Children’s Body Shape Objective?	Yes	0.1578	0.0688	0.0229	0.2926	0.0218
	No (ref.)					
Does Childhood Obesity Need Early Prevention?	No	−0.3101	0.1613	−0.6262	0.00600	0.0545
	Yes but it’s too early	0.0799	0.1201	−0.1555	0.3153	0.5058
	Yes, we should start prevention now (ref.)					
Frequency of Teaching Children about Healthy Diet	Always	0.1724	0.2455	−0.3088	0.6536	0.4826
	Often	0.1763	0.1694	−0.1558	0.5083	0.2982
	Seldom	−0.0805	0.2078	−0.4878	0.3267	0.6983
	Never	−0.2804	0.5459	−1.3503	0.7895	0.6075
	Occasionally (ref.)					

Note: SE—standard error of mean and 95% CI—95% confidence intervals.
